# Emerging Evidence on Balneotherapy and Thermal Interventions in Post-COVID-19 Syndrome: A Systematic Review

**DOI:** 10.3390/healthcare13020096

**Published:** 2025-01-07

**Authors:** Elisabetta Ferrara, Manela Scaramuzzino, Giovanna Murmura, Gianmaria D’Addazio, Bruna Sinjari

**Affiliations:** 1 Department of Human Sciences, Law, and Economics, Telematic University Leonardo Da Vinci, UNIDAV, Torrevecchia Teatina, 66100 Chieti, Italy; 2 Medical Thermal Center of Saturnia, 58014 Saturnia, Italy; manela.scaramuzzino@gmail.com; 3 Unit of Prostodontics, Department of Innovative Technologies in Medicine and Dentistry, University “G. D’Annunzio” Chieti-Pescara, 66100 Chieti, Italy; g.murmura@unich.it (G.M.); gianmaria.daddazio@unich.it (G.D.); bruna.sinjari@unich.it (B.S.)

**Keywords:** long COVID-19 syndrome, post-acute COVID-19 sequelae, balneotherapy, thermal therapy, spa therapy, respiratory rehabilitation, hydrotherapy

## Abstract

Background: Post-COVID-19 syndrome affects 10–60% of SARS-CoV-2 survivors. While conventional treatments show limited efficacy, emerging evidence suggests the potential benefits of balneotherapy in managing persistent symptoms. We aimed to systematically evaluate the efficacy and safety of balneotherapy and thermal treatment interventions in treating post-COVID-19 syndrome. Methods: We conducted a systematic review following PRISMA guidelines, searching major databases through 31 January 2024. Eligible studies included randomized controlled trials, observational studies, and pilot studies investigating thermal spa treatments for adult post-COVID-19 patients. Results: Analysis of six eligible studies (n = 617) demonstrated significant therapeutic benefits. The largest cohort (n = 159) showed 47% reduction in fatigue and 48% reduction in muscle pain (*p* < 0.01). Comprehensive spa therapy achieved complete symptom resolution in one-third of the participants. Combined spa-ubiquinol therapy improved metabolic function (*p* < 0.05). All interventions demonstrated favorable safety profiles. Conclusions: Preliminary evidence suggests balneotherapy effectively ameliorates multiple post-COVID-19 symptoms, particularly fatigue, muscle pain, and exercise intolerance. While safety profiles appear favorable, larger randomized controlled trials with standardized protocols are needed to establish definitive therapeutic recommendations.

## 1. Introduction

The COVID-19 pandemic has precipitated unprecedented global health challenges, with over 771 million confirmed cases and more than 6.9 million fatalities worldwide [1,2,3]. While initial efforts primarily focused on managing the acute phase of the disease, emerging evidence has revealed that 10–60% of COVID-19 survivors experience persistent symptoms long after the resolution of the acute infection [4,5]. The post-acute sequelae of SARS-CoV-2 infection, commonly referred to as ‘long COVID’, have emerged as a significant clinical entity requiring comprehensive therapeutic approaches [6]. Long COVID is characterized by a diverse array of symptoms persisting for weeks to months after the acute phase of COVID-19, affecting multiple organ systems. These symptoms include fatigue, dyspnea, cognitive impairment, cardiovascular complications, and gastrointestinal issues [6,7,8]. The complex pathophysiology involves persistent inflammation, immune dysregulation, and autonomic dysfunction [9,10,11]. Recent evidence demonstrates that traditional therapeutic approaches alone may not be sufficient to address the full spectrum of symptoms experienced by patients. Balneotherapy has demonstrated significant therapeutic efficacy across various chronic conditions, particularly in managing inflammatory and musculoskeletal disorders. Recent randomized controlled trials have provided strong evidence for its clinical benefits. Karagülle et al. [12] demonstrated that a 2-week spa therapy program, as an adjunct to usual pharmacological therapy, provided sustained clinical improvements in rheumatoid arthritis patients for up to 6 months, with significant reductions in disease activity scores and inflammatory markers. Similarly, Ozkuk et al. [13] reported significant improvements in pain, disability, and quality of life measures in patients with chronic shoulder pain following balneotherapy interventions, with benefits persisting for up to 3 months post-treatment. These findings are particularly relevant for post-COVID-19 rehabilitation, given the similar inflammatory and musculoskeletal manifestations observed in long COVID patients. The therapeutic mechanisms of balneotherapy extend beyond simple symptom management. Perez-Fernandez et al. [14] demonstrated that mineral-medicinal water baths can produce significant physiological effects through multiple pathways, including enhanced pain thresholds, improved cardiovascular function, and modulation of inflammatory responses. Their randomized crossover trial showed sustained therapeutic benefits lasting up to 8 months post-intervention, suggesting potential long-term efficacy for chronic conditions. These mechanistic insights are particularly relevant for post-COVID-19 syndrome, where multiple system involvement necessitates comprehensive therapeutic approaches. This systematic review aimed to synthesize the available evidence on the efficacy of thermal treatments for post-COVID-19 symptoms, considering both established mechanistic pathways and emerging clinical data. By examining the intersection between traditional balneotherapy evidence and specific post-COVID-19 applications, we seek to provide a comprehensive analysis of this therapeutic approach’s potential in managing long COVID symptoms.

## 2. Materials and Methods

### 2.1. Protocol and Registration

This systematic review was conducted in accordance with the Preferred Reporting Items for Systematic Reviews and Meta-Analyses (PRISMA) guidelines [15]. The protocol was registered in the PROSPERO database (Registration number: CRD42024592036).

### 2.2. Search Strategy

A systematic literature search was performed in PubMed, Scopus, and Web of Science databases through 31 January 2024. The search strategy combined Medical Subject Headings (MeSHs) terms and free-text keywords related to two main concepts: post-COVID-19 condition and thermal interventions. For PubMed, MeSH terms included “COVID-19” AND (“therapeutics” OR “rehabilitation”), combined with free-text terms “post-acute”, “long COVID”, “balneotherapy”, “thermal therapy”, and “spa therapy”. Search strings were adapted for Scopus and Web of Science according to their specific indexing requirements while maintaining conceptual equivalence. Boolean operators and field limiters were applied to ensure comprehensive retrieval of the relevant literature. Additional relevant studies were identified through manual screening of reference lists from the included papers and pertinent reviews. Study selection and data extraction were performed independently by two reviewers using Covidence systematic review software (www.covidence.org, Veritas Health Innovation, Melbourne, Australia) for duplicate removal and screening process. Given the emerging nature of research in post-COVID balneotherapy, all available studies meeting the above criteria were included, regardless of sample size, to ensure comprehensive coverage of the available evidence.

### 2.3. Eligibility Criteria

Studies were selected based on the following PICOS criteria, summarized in Table 1.

The minimum treatment duration of 5 days was established based on the predominant symptom approach in post-COVID-19 patients. While standard balneotherapy protocols typically require 9–14 days for optimal therapeutic benefits in chronic conditions [17] with effects doubling exponentially at 14 days, we adopted a more flexible criterion considering the novelty of post-COVID-19 syndrome and the varying severity and presentation of fatigue as its primary symptom.

### 2.4. Data Extraction and Synthesis

Data extraction was performed independently by two reviewers (M.S. and G.M.) using a standardized, pre-piloted form. Extracted information included study characteristics, participant demographics, intervention details, outcome measures, key findings, and adverse events. A narrative synthesis approach, following the Synthesis Without Meta-analysis (SWiM) guidelines [18], was adopted due to the anticipated heterogeneity in interventions and outcomes. The synthesis considered various factors, including treatment modalities, patient characteristics, and study design features.

### 2.5. Risk of Bias Assessment

We assessed methodological quality using validated tools appropriate for each study design. For randomized controlled trials, we applied the Revised Cochrane risk-of-bias tool (RoB 2), which evaluates five domains: randomization process, deviations from intended interventions, missing outcome data, outcome measurement, and selective reporting. For non-randomized studies (including retrospective, prospective observational, and cohort studies), we employed the Risk Of Bias In Non-Randomized Studies of Interventions (ROBINS-I) tool. ROBINS-I assesses seven domains: confounding, participant selection, intervention classification, deviations from intended interventions, missing data, outcome measurement, and selective reporting. Two reviewers (M.S. and G.D.) independently conducted the risk of bias assessment for each included study. Before formal assessment, the reviewers underwent standardized training and performed calibration exercises on a sample of similar studies not included in the review. Discrepancies in assessments were resolved through discussion, with a third reviewer (B.S.) being consulted when consensus could not be reached. For each study, we categorized the overall risk of bias as low, moderate, or serious based on domain-specific assessments, following the tools’ guidance documents.

## 3. Results

**Figure 1:**
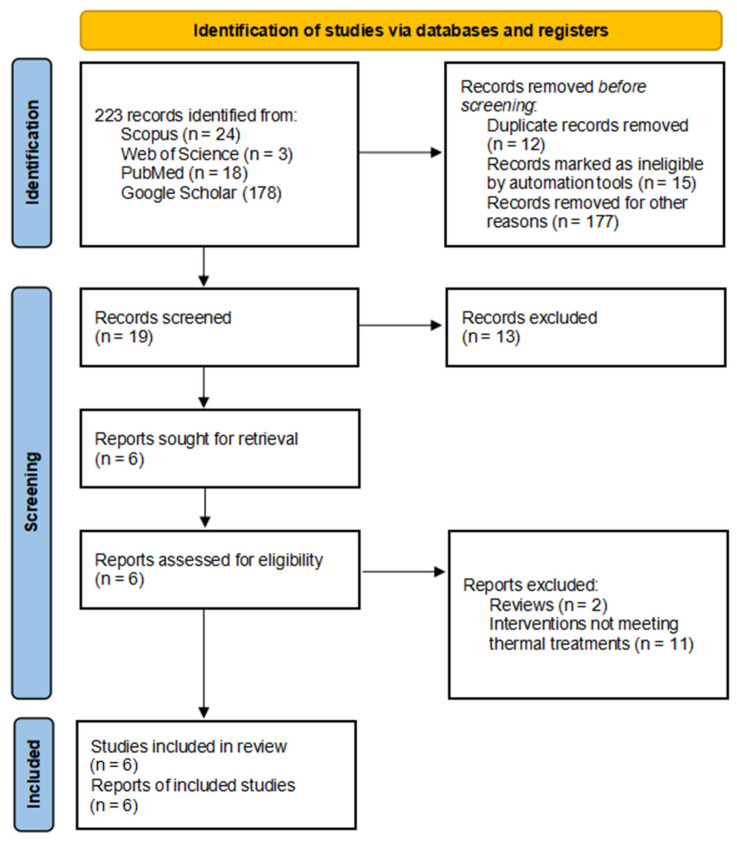
PRISMA Flow Chart.

The systematic search yielded 223 potentially relevant records: 24 from Scopus, 3 from Web of Science, 18 from PubMed, and 178 from Google Scholar. Records removed before screening included 12 duplicate records, 15 records marked as ineligible by automation tools, and 177 records not related to balneotherapy/thermal treatment protocols. Nineteen unique citations remained for title and abstract screening. Of these, 13 records were excluded, resulting in 6 reports sought for retrieval. All six reports were assessed for eligibility, and all six were included in the final qualitative synthesis (Figure 1).

The characteristics and main findings of the included studies are summarized in Table 2.

### 3.1. Effects on Primary Outcomes

The therapeutic impact of balneotherapy on symptom severity was consistently reported across studies. Among specific symptoms, Costantino et al. [19] documented significant improvements in chronic fatigue and muscle pain, with reductions of 47% and 48%, respectively. Respiratory function improvements were particularly notable in Onik et al.’s study [21], which reported a 62% reduction in mMRC dyspnea scale scores (*p* < 0.0001). The quality of life measurements, assessed through SF-36 in Costantino et al.’s study [19], demonstrated meaningful improvements in both physical and emotional domains, with women showing more consistent improvements across all quality of life dimensions compared to men.

### 3.2. Treatment Response Patterns

The included studies employed varying treatment durations, from 10 days [23] to 3 months [20]. Crucianelli et al.’s randomized trial demonstrated significant improvements in respiratory function and inflammatory markers with sulfur thermal water inhalations compared to placebo [20]. Combination approaches, such as those employed by Ponikowska et al. [22], yielded particularly promising results, with 30.3% of patients achieving complete symptom resolution through a comprehensive program combining balneotherapy with other therapeutic modalities.

### 3.3. Treatment Adherence and Patient Satisfaction

Treatment adherence rates across studies ranged from 89% to 95%. Patient satisfaction metrics, assessed via standardized questionnaires in three studies, demonstrated high satisfaction levels (mean satisfaction scores > 8/10) with particularly positive feedback regarding symptom improvement and therapeutic environment. Costantino et al. [19] reported that 87% of participants rated their treatment experience as ’satisfactory’ or ’highly satisfactory’, while Ponikowska et al. [22] documented an 85% positive response rate for overall treatment satisfaction.

### 3.4. Safety and Tolerability

The safety profile of balneotherapy was consistently favorable across studies. Crucianelli et al. [20] reported no adverse events in their randomized trial, while other studies reported only mild and transient side effects. The comprehensive health resort approaches were well-tolerated across different age groups and patient populations.

### 3.5. Results of Risk of Bias Assessment

The risk of bias assessment revealed varying levels of methodological quality across the included studies (Table 3). The risk of bias assessment revealed varying levels of methodological quality across the included studies. The randomized controlled trial by Crucianelli et al. [20] demonstrated the lowest risk of bias among all studies, with low risk in four domains and some concerns only in the selection of reported results. For the non-randomized studies, which comprise the majority of our sample, the risk of bias ranged from low to moderate. Costantino et al. [19], Onik et al. [21], and Ponikowska et al. [22] were assessed as having a moderate overall risk of bias, primarily due to concerns in the confounding domain. Shchikota et al. [23] and Grishechkina et al. [24] were evaluated as having a low overall risk of bias, showing better performance across most domains. Common limitations across most non-randomized studies included potential confounding factors, with all studies rated as having moderate risk in this domain. However, these studies generally performed well in domains such as selection of participants, classification of interventions, and deviations from intended interventions, which were consistently rated as low risk. The domains of missing data and measurement of outcomes showed some variability across studies. Notably, Onik et al. [21] and Shchikota et al. [23] had no information available for the missing data domain, while the other studies were assessed as having low risk in this area. The measurement of outcomes was rated as a moderate risk for Costantino et al. [19] but low risk for the other studies. Overall, while some methodological concerns were identified, particularly regarding confounding in non-randomized studies, the majority of the included studies demonstrated reasonably robust methodological quality, with low to moderate overall risk of bias.

## 4. Discussion

The pathophysiological complexity of post-COVID-19 syndrome requires therapeutic approaches capable of addressing multiple system involvement. Balneotherapy, through its diverse mechanisms of action, demonstrates a particular relevance in this context. The immunomodulatory effects of thermal therapy, documented by Gálvez et al. [25] include regulation of pro-inflammatory cytokines and enhancement of cell-mediated immunity, directly addressing the persistent inflammatory state characteristic of post-COVID-19 syndrome.

The therapeutic benefits of aquatic interventions extend beyond immunomodulation through their distinct biomechanical properties. Water immersion creates a unique therapeutic environment characterized by hydrostatic pressure and buoyancy. The hydrostatic pressure, ranging from 0.35 to 1.0 atmospheres during immersion, enhances cardiovascular function through increased venous return and stroke volume [26], particularly beneficial for patients experiencing post-COVID fatigue and exercise intolerance.

The hydrodynamic properties of aquatic environments offer distinct advantages for musculoskeletal rehabilitation. Water’s buoyancy reduces effective body weight by approximately 90% when immersed to neck level, while simultaneously providing multidirectional resistance that increases proportionally with movement velocity [27]. This combination creates an optimal rehabilitative medium that enables progressive muscle strengthening while protecting reconditioned joints. The resistance patterns allow for natural autoregulation of exercise intensity—a critical feature for patients with varying levels of fatigue and exercise tolerance. This graduated loading approach facilitates the recovery of strength and endurance in patients with post-COVID muscle weakness, while the reduced gravitational stress enables movement patterns that might be impossible under full terrestrial conditions.

Furthermore, these biomechanical properties demonstrate particular efficacy in addressing post-exertional malaise, a hallmark feature of long COVID. The controlled resistance environment, coupled with enhanced cardiovascular function through hydrostatic pressure effects, provides a comprehensive approach to reconditioning those accounts for both peripheral and central aspects of post-COVID fatigue syndrome.

This finding could have important implications for understanding the pathophysiology of PASC and the mechanisms by which balneotherapy and thermal medicine interventions may exert their effects. Research has elucidated several key components in the effective treatment of long COVID, encompassing self-management techniques, physical rehabilitation protocols, and targeted pharmacological interventions. Emerging evidence suggests that balneotherapy and associated hydrotherapeutic interventions may play a significant role in this multifaceted management of post-acute sequelae of SARS-CoV-2 infection (PASC) [19,20,21,22,23,24,25]. Within this comprehensive framework, balneotherapy emerges as a promising adjunctive therapy, offering unique benefits that complement existing treatment modalities. Its capacity to address both physical and immunological aspects of long COVID makes it a compelling area for clinical investigation. Balneotherapy has demonstrated significant efficacy in improving respiratory function and overall well-being in various chronic respiratory conditions, particularly through enhancement of mucociliary clearance, reduction in inflammatory cytokine production, and preservation of lung elastic properties in patients with chronic obstructive pulmonary disease (COPD) [28,29,30,31,32]. Given the similarities in respiratory challenges faced by COPD and long COVID patients, these findings provide a strong rationale for exploring balneotherapy in post-COVID-19 rehabilitation. Water-based exercise training, a key component of hydrotherapy, offers a particularly promising approach for long COVID patients experiencing fatigue and musculoskeletal weakness [33,34,35,36,37]. This low-impact form of therapy has demonstrated improvements in exercise capacity and health-related quality of life, addressing barriers posed by comorbidities such as obesity, which are often exacerbated in long COVID cases [38]. Moreover, the anti-bacterial and anti-inflammatory properties of sulfur-rich mineral waters may reduce the risk of respiratory infections, particularly beneficial for patients with increased susceptibility to respiratory complications [39,40,41]. The spa environment associated with balneotherapy contributes to alleviating psychological stress, which is known to have immunosuppressive effects, while simultaneously modulating both cell-mediated and humoral immunity [25,42]. Studies have demonstrated increases in the levels and activity of immune cells, such as neutrophils and monocytes, following balneotherapy interventions [23,24,25]. For instance, radon bath applications have been associated with a small but sustained increase in monocyte levels, while in vitro research has shown that sulfur water enhances the short-term survival capacity of neutrophils. Clinical trials have reported reductions in pro-inflammatory cytokines, including TNF-α and IL-1β, after balneotherapy treatments [43,44]. This anti-inflammatory effect is particularly relevant for individuals recovering from COVID-19, given the significant role of inflammatory processes in the disease’s pathophysiology. This finding could have important implications for understanding the mechanisms by which balneotherapy and thermal medicine interventions exert their effects. Balneotherapy has demonstrated significant efficacy in improving respiratory function and overall well-being in various chronic respiratory conditions [38,43]. Antonelli et al. [29] highlighted several mechanisms by which balneotherapy and hydrotherapy ameliorate respiratory dysfunction. These therapies enhance mucociliary clearance, reduce inflammatory cytokine production, and preserve lung elastic properties in patients with chronic obstructive pulmonary disease (COPD). Given the similarities in respiratory challenges faced by COPD and long COVID patients, these findings provide a strong rationale for post-COVID-19 rehabilitation [32].

Water-based exercise training, a key component of hydrotherapy, offers a particularly promising approach for patients experiencing fatigue and musculoskeletal weakness [45]. This low-impact therapy has demonstrated improvements in exercise capacity and health-related quality of life, addressing barriers posed by comorbidities such as obesity, which are often exacerbated in long COVID cases [46].

This therapeutic approach is further supported by Shchikota A.M. et al. [23], who examined various hydrotherapy modalities in outpatient rehabilitation for the post-COVID syndrome. Their investigation of 160 participants, divided into three intervention groups and a control group, administered treatments over a 10-day period. The comprehensive evaluation included clinical symptoms, cardiopulmonary function, hormonal status, allostatic load, and psychological parameters. All intervention groups demonstrated significant improvements across multiple domains compared to controls, including symptom reduction, enhanced cardiopulmonary function, and positive changes in hormonal and psychological profiles. The observed decrease in the allostatic load suggested a reduced physiological stress. While each modality showed unique benefits, the results confirmed hydrotherapy’s value in post-COVID rehabilitation.

**Figure 2:**
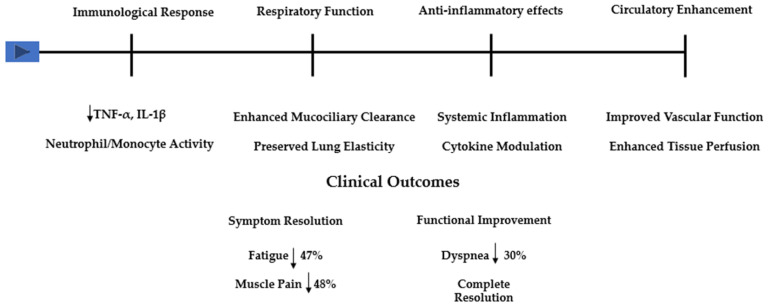
Schematic representation of balneotherapy’s therapeutic mechanisms and clinical outcomes in post-COVID-19 syndrome. The upper section illustrates the four main pathophysiological pathways: immunological modulation (including effects on TNF-α, IL-1β, and neutrophil/monocyte activity), respiratory function enhancement (through mucociliary clearance improvement and lung elasticity preservation), anti-inflammatory effects (via systemic inflammation reduction and cytokine modulation), and circulatory enhancement (improving vascular function and tissue perfusion). The lower section summarizes key clinical outcomes from the included studies, demonstrating symptom resolution rates for fatigue (47%) and muscle pain (48%), alongside functional improvements, including dyspnea reduction (62%) and complete symptom resolution in 30.3% of cases.

The anti-bacterial and anti-inflammatory properties of sulfur-rich mineral waters provide additional benefits, particularly for patients with increased susceptibility to respiratory complications [1,4,32,33]. This aspect reinforces the comprehensive approach needed for long COVID management. The therapeutic mechanisms of balneotherapy in post-COVID-19 syndrome operate through multiple, complementary pathways (Figure 2).

These pathways, supported by both established research in chronic conditions and emerging evidence in post-COVID-19, demonstrate how balneotherapy’s effects extend beyond simple symptom management to address underlying pathophysiological processes. As illustrated in Figure 2, the immunological response includes modulation of both cellular and humoral immunity, with documented effects on neutrophil and monocyte activity, and a reduction in pro-inflammatory cytokines, such as TNF-α and IL-1β. The respiratory benefits, evident in the improvement in mucociliary clearance and preservation of lung elasticity, directly address key pathophysiological alterations seen in post-COVID-19 syndrome. These mechanisms, complemented by systemic anti-inflammatory effects and enhanced circulatory function, explain the broad range of clinical improvements observed across studies.

The quantitative findings from our included studies demonstrate notable improvements in fatigue (47% reduction), muscle pain (48% reduction), and dyspnea (62% reduction in mMRC scores). The achievement of complete symptom resolution in 30.3% of treated patients, as reported by Ponikowska et al. [22], suggests significant clinical recovery potential. This mechanistic framework explains balneotherapy’s suitability for post-COVID-19 rehabilitation through its simultaneous action on multiple pathophysiological aspects.

Crucianelli et al. [20] provided compelling evidence through their study of sulfur thermal water (STW) inhalations compared with placebo treatments. The improvements in the St. George’s Respiratory Questionnaire scores and 6-minute walk test distances demonstrated enhanced quality of life and functional capacity in the STW group.

These outcomes were consistent with previous research on hydrotherapy for respiratory conditions, extending its clinical application to post-COVID sequelae. The reduction in inflammatory biomarkers, particularly IL-6 and IL-1β, in the STW group represents a key mechanism of action, attributed to the hydrogen sulfide (H_2_S) present in thermal water, known for its anti-inflammatory and antiviral properties. The exploration of nasal microbiome changes further elucidated the mechanisms of STW inhalations, with observed increases in certain bacterial genera suggesting beneficial modifications to respiratory microbiome composition.

These findings align with studies by Costantino et al. [19], which demonstrated the efficacy of thermal therapy in alleviating persistent post-COVID-19 symptoms, with a notable gender disparity showing enhanced improvements in female participants’ quality of life measures.

The study by Ponikowska et al. [22] reinforced these results through a comprehensive health resort program. Their research, involving 33 patients treated with a multifaceted approach, including dietary treatment, kinesiotherapy, balneotherapy, physical therapy, and oxygen therapy, demonstrated significant clinical improvements. The complete resolution of symptoms in one-third of the patients and enhanced physical capacity validate the effectiveness of structured rehabilitation programs in addressing persistent fatigue and reduced physical function. These results align with other investigations into non-pharmacological management of post-COVID conditions, highlighting the value of comprehensive treatment approaches that address the multisystem impact of long COVID.

Onik et al. [21] further confirmed the efficacy of health resort treatment for long COVID symptoms. The significant reduction in mMRC scale scores and improvements in subjective symptom assessments demonstrate that this comprehensive approach, combining balneotherapy, exercise, and physical medicine modalities, provides meaningful relief for patients. The comparable efficacy across different age groups and between sexes indicates broad applicability to diverse patient populations. However, the lack of improvement in tachycardia symptoms warrants further investigation and suggests a need for targeted cardiovascular interventions. The inhalation of mineral-rich vapors enhances mucociliary clearance and overall respiratory function, addressing the altered diffusion capacity and restrictive patterns in post-COVID-19 patients [40]. Firouzi et al. [47] demonstrated that mineral nanoparticles (MNPs) from thermal spring water exhibit antiviral effects against SARS-CoV-2 in vitro and in clinical settings. The unique physicochemical properties of these MNPs, including their small size and high surface area, facilitate interaction with viral proteins, potentially inhibiting viral entry and replication. Further research in larger clinical studies will validate these effects. Cold water immersion increases leukocytes, granulocytes, circulating levels of interleukin-6, and natural killer cell activity. Daily brief cold stress enhances both numbers and activity of peripheral cytotoxic T-lymphocytes and NK cells, major effectors of adaptive and innate tumor immunity. The holistic nature of balneotherapy aligns with guidelines from the Italian Thermal Medicine Foundation [48]. While safety adaptations, including stringent hygiene measures and social distancing, have modified the traditional spa environment, the establishment of dedicated “COVID Units” has enabled specialized care for post-COVID-19 patients.

## 5. Future Directions

While the current evidence suggests promising potential for balneotherapy and hydrotherapy in managing long COVID symptoms, several key areas require further investigation. Firstly, large-scale, randomized controlled trials are needed to definitively establish the efficacy of these interventions across diverse patient populations. Secondly, longitudinal studies should be conducted to assess the long-term benefits and potential risks of these therapies. Thirdly, research should focus on optimizing treatment protocols, including the ideal duration, frequency, and combination of different balneotherapy modalities. Additionally, mechanistic studies are crucial to elucidate the precise physiological pathways through which these therapies exert their effects, particularly in the context of post-COVID-19 pathophysiology. Finally, cost-effectiveness analyses would be beneficial to evaluate the feasibility of integrating these therapies into standard care protocols for long COVID management.

## 6. Conclusions

The evidence suggests that these interventions may offer benefits in addressing multiple aspects of post-COVID-19 syndrome, including respiratory function, fatigue, and overall quality of life. The multifaceted nature of these therapies aligns well with the complex, systemic manifestations of long COVID. However, while the initial findings are encouraging, they must be interpreted with caution due to the limitations in the study designs and the evolving nature of COVID-19 safety protocols in thermal facilities. The integration of balneotherapy into comprehensive rehabilitation programs for long COVID patients warrants further investigation through rigorous, large-scale clinical trials. As our understanding of long COVID continues to evolve, balneotherapy may emerge as a valuable component of a holistic approach to patient care, potentially offering relief and improved outcomes for those struggling with the long-term effects of SARS-CoV-2 infection.

## Figures and Tables

**Table 1:** PICOS Criteria.

Criterion	Description
Population	Adults (≥18 years) with confirmed post-acute COVID-19 syndrome. The 12-week minimum interval from initial SARS-CoV-2 infection was adopted following WHO criteria for post-COVID-19 condition definition [6]
Intervention	Balneotherapy, thermal therapy, or thermal spa treatments. The minimum treatment duration of 5 days was established based on previous systematic reviews demonstrating this as the minimum period necessary to achieve therapeutic effects in balneotherapy protocols [16]. All interventions required supervision by healthcare professionals and clear documentation of water composition and treatment protocols
Outcomes	Primary: Changes in symptom severity (validated scales) Quality of life measures Functional capacity assessments Secondary: Physiological parameters Adverse events Treatment adherence Patient satisfaction
Study design	Randomized controlled trials, non-randomized controlled trials, prospective and retrospective cohort studies, and case-control studies

**Table 2:** Characteristics and Findings of Included Studies.

Author (Year)	Study Design and Sample	Intervention Details	Duration and Assessment	Primary Outcomes	Key Findings
Costantino et al. (2024) [19]	Prospective observational; n = 159 (87 symptomatic)	Comprehensive spa therapy program, including balneotherapy and/or inhalation therapy	2-week therapeutic spa cycle; Follow-up assessments at completion	Post-COVID-19 symptom severity (VAS) Quality of Life (SF-36)	47% reduction in fatigue 48% reduction in muscle pain Enhanced quality of life in physical and emotional domains Women showed more consistent improvement High treatment satisfaction reported Protective effect of vaccination observed
Crucianelli et al. (2024) [20]	Double-blind, randomized pilot trial; n = 30 (15 per group)	Active: Sulfur thermal water inhalations Control: Sterile distilled water inhalations Daily treatments	12 consecutive days; Assessments at baseline, 14 days, 3 months	Clinical symptoms (SBQ-LC, SGRQ) Pulmonary function Inflammatory markers Microbiome analysis	Significant SGRQ score improvement Enhanced 6 MWT performance Reduced inflammatory markers Beneficial microbiome changes No adverse events reported
Onik et al. (2024) [21]	Retrospective study; n = 122 (71 F, 51 M)	Comprehensive health resort treatment: Balneotherapy Exercise therapy Physical medicine Health education	Mean 24.59 ± 6.38 days	Cardiopulmonary symptoms (0–10 scale) mMRC dyspnea scale	62% reduction in mMRC scores (*p* < 0.0001) Greater improvement in women Age-independent efficacy Similar benefits across treatment modalities
Ponikowska et al. (2023) [22]	Prospective intervention; n = 33 (19 F, 14 M)	Multimodal program: Balneotherapy Kinesiotherapy Physical therapy Oxygen therapy Dietary intervention	14–28 days; 5 procedures daily	Long COVID symptoms Physical capacity (VO_2_ max) Depression assessment	30.3% complete symptom resolution Significant symptom reduction (*p* < 0.001) 68.4% achieved normal capacity Improvements in fatigue, cognition, pain
Shchikota et al. (2023) [23]	Prospective cohort with parallel groups; n = 160 (74 M, 86 F)	Four treatment arms: 1. Chloride-sodium baths 2. Whirlpool baths 3. “Biolong” baths 4. Control group All with exercise therapy	10-day hydrotherapy course	Clinical symptoms ECG parameters Pulmonary function Exercise capacity Hormonal status Psychological status	Significant symptom improvement in all intervention groups Enhanced lung function Improved hemodynamics Reduced stress markers Better psychological outcomes
Grishechkina et al. (2023) [24]	Prospective cohort; n = 113 (120 initial, 7 dropouts)	Four rehabilitation approaches: Experimental: Comprehensive program CG1: Eastern medicine CG2: Standard rehabilitation CG3: Self-directed exercise	7–15 sessions; 6-month follow-up	Hospital admissions Specialist consultations Clinical outcomes Disability onset	85.7% reduced hospital admissions Fewer specialist consultations Better symptom control No new disabilities Cost-effective outcomes

Abbreviations: VAS = Visual Analogue Scale; SF-36 = Short Form 36 Health Survey; SBQ-LC = Specific Breathing Questionnaire for Long COVID; SGRQ = St. George’s Respiratory Questionnaire; 6 MWT = Six-Minute Walk Test; mMRC = modified Medical Research Council; ECG = Electrocardiogram; F = Female; M = Male; CG = Comparison Group. Note: Statistical significance (*p*-values) reported where available in original studies.

**Table 3:** Quality Assessment of Included Studies.

Study	Design	Tool	Domain 1	Domain 2	Domain 3	Domain 4	Domain 5	Domain 6	Domain 7	Overall Risk
Costantino et al. [19]	Prospective observational	ROBINS-I	Confounding: Moderate	Selection of participants: Low	Classification of interventions: Low	Deviations from intended interventions: Low	Missing data: Low	Measurement of outcomes: Moderate	Selection of the reported result: Low	Moderate
Crucianelli et al. [20]	RCT	RoB 2	Randomization process: Low	Deviations from intended interventions: Low	Missing outcome data: Low	Measurement of the outcome: Low	Selection of the reported result: Some concerns	N/A	N/A	Low
Onik et al. [21]	Retrospective	ROBINS-I	Confounding: Moderate	Selection of participants: Low	Classification of interventions: Low	Deviations from intended interventions: Low	Missing data: No information	Measurement of outcomes: Low	Selection of the reported result: Low	Moderate
Ponikowska et al. [22]	Prospective intervention	ROBINS-I	Confounding: Moderate	Selection of participants: Low	Classification of interventions: Low	Deviations from intended interventions: Low	Missing data: Low	Measurement of outcomes: Low	Selection of the reported result: Moderate	Moderate
Shchikota et al. [23]	Prospective cohort	ROBINS-I	Confounding: Moderate	Selection of participants: Low	Classification of interventions: Low	Deviations from intended interventions: Low	Missing data: No information	Measurement of outcomes: Low	Selection of the reported result: Moderate	Low
Grishechkina et al. [24]	Prospective cohort	ROBINS-I	Confounding: Moderate	Selection of participants: Low	Classification of interventions: Low	Deviations from intended interventions: Low	Missing data: Low	Measurement of outcomes: Low	Selection of the reported result: Moderate	Low

## Data Availability

Not applicable.
